# Effects of Different Initial pH Conditions on the Antioxidant Capacity and Lipidomic Profiles of *Samsoniella hepialid*

**DOI:** 10.3390/jof12050367

**Published:** 2026-05-16

**Authors:** Yan Tong, Chuyu Tang, Bing Jia, Haoxu Tang, Jinxuan Yan, Yuling Li, Xiuzhang Li

**Affiliations:** State Key Laboratory of Plateau Ecology and Agriculture, Qinghai Academy of Animal and Veterinary Sciences, Qinghai University, Xining 810016, China

**Keywords:** *Samsoniella hepiali*, initial pH, antioxidant capacity, lipidomics, sphingolipid metabolism

## Abstract

*Samsoniella hepiali* produces an array of pharmacologically valuable metabolites, but how environmental pH regulates its antioxidant system and lipid metabolism during submerged fermentation remains unclear. This study aimed to investigate the effects of different initial culture pH values (pH 4, 5, 6, and 7) on the antioxidant capacity and lipidomic metabolism of *S. hepiali*. The results demonstrated that at pH 5, the activities of peroxidase (POD) and superoxide dismutase (SOD), the contents of total phenolics (TP) and flavonoids, the scavenging rates of DPPH• and •OH, and the total antioxidant capacity all peaked. Conversely, the level of glutathione (GSH) reached its maximum at pH 6 (0.69 ± 0.014 μmol/g). Lipidomic analysis identified a total of 404 lipid molecular species, mainly TG, PE, and DG. Comparative analysis among pH 4 vs. pH 5, pH 6 vs. pH 5, and pH 7 vs. pH 5 revealed 27 core DALs belonging to 11 lipid subclasses, most of which were upregulated at pH 5. KEGG pathway enrichment analysis further revealed that sphingolipid metabolism was the sole core co-enriched pathway under different pH conditions. Particularly at pH 5, key signaling lipids, such as ceramides, underwent pronounced targeted accumulation. This study elucidates the molecular adaptation mechanisms of medicinal fungi in response to pH variation from a lipidomic perspective. It provides a basis for optimizing fermentation conditions to enhance antioxidant activity and functional lipid production.

## 1. Introduction

*Ophiocordyceps sinensis* is a highly valued traditional medicinal fungus in China. It contains diverse bioactive compounds, including polysaccharides, terpenoids, alkaloids, and phenolics, with anti-inflammatory, antitumor, and immunomodulatory activities [1,2,3,4,5,6]. However, wild *O. sinensis* resources are declining because of its specialized parasitic life cycle, climate change, and persistent overharvesting [7,8,9,10]. *Samsoniella hepiali* is an entomopathogenic fungus that can infect *Thitarodes* hosts [11]. It has been demonstrated to synthesize active constituents highly analogous to those of *O*. *sinensis*, including polysaccharides, mannitol, adenosine, specific lipids, and flavonoids [12,13,14]. Consequently, the highly efficient cultivation of *S. hepiali* via submerged fermentation technology has emerged as a promising strategy to alleviate the shortage of wild *O. sinensis*.

During fermentation, environmental factors strongly affect mycelial growth and secondary metabolic networks [15,16]. Among them, pH is a key factor influencing antioxidant capacity. Previous studies have shown that pH affects the yield and activity of antioxidant compounds in *Spirulina* [17] and can also affect the antioxidant capacity of polysaccharides from *Cordyceps militaris* [18]. Lipids serve not only as the structural foundation of the cellular membrane barrier and intracellular energy reservoirs but also as crucial signaling hubs that govern cell growth, differentiation, and environmental adaptability [19,20,21]. The pH of the medium significantly influences both the lipid yields [22,23] and lipid composition of fungi [24]. Recent advances in high-resolution mass spectrometry have made UPLC-MS/MS-based lipidomics a powerful approach for profiling metabolic responses to environmental changes [25]. However, the effects of pH on antioxidant capacity and lipid metabolism in *S. hepiali* remain unclear.

Accordingly, this study examined *S. hepiali* cultured under different initial pH conditions. Enzymatic and non-enzymatic antioxidant responses were evaluated, and UPLC-MS/MS-based lipidomics was applied to characterize pH-dependent changes in mycelial lipid metabolism. These results provide new insight into the molecular basis of pH adaptation in medicinal fungi.

## 2. Materials and Methods

### 2.1. Fungal Strain and Cultivation Conditions

The *S. hepiali* strain used in this study was provided by the Cordyceps Laboratory, Academy of Animal Science and Veterinary Medicine, Qinghai University (Xining, China). *S. hepiali* was cultured in a liquid potato dextrose medium containing 20% potato extract (Qinghai Libaochang Trading Co., Ltd., Xining, China), 2% glucose (Qinghai Libaochang Trading Co., Ltd., Xining, China), 0.3% peptone (Qinghai Libaochang Trading Co., Ltd., Xining, China), 0.2% KH_2_PO_4_ (Qinghai Libaochang Trading Co., Ltd., Xining, China), and 0.02% MgSO_4_ (Qinghai Libaochang Trading Co., Ltd., Xining, China). The initial pH of the basal medium was adjusted to 4, 5, 6, and 7 using NaOH (Qinghai Libaochang Trading Co., Ltd., Xining, China) and HCl (Qinghai Libaochang Trading Co., Ltd., Xining, China). After autoclaving, the pH of each medium was remeasured to ensure consistency with the experimental design. The activated strain was inoculated into the media with varying pH levels and incubated in a rotary shaker at 18 °C and 135 rpm for 7 days (Shanghai Zhichu Instrument Co., Ltd. Shanghai, China). The cultures were then centrifuged at 10,000× *g* for 20 min at 4 °C to harvest the mycelia. The resulting mycelial pellets were washed with sterile water and immediately stored at −80 °C for subsequent analyses.

### 2.2. Determination of Antioxidant Capacity

To comprehensively evaluate the antioxidant potential of the mycelia, this study measured the total antioxidant capacity via ferric reducing antioxidant power (FRAP) and 2,2′-azinobis(3-ethylbenzothiazoline-6-sulfonic acid) (ABTS) assays, the scavenging rates of 2,2-diphenyl-1-picrylhydrazyl radical (DPPH•) and hydroxyl radicals (•OH), and the activities of key antioxidant enzymes, including catalase (CAT), glutathione peroxidase (GSH-Px), peroxidase (POD), and superoxide dismutase (SOD). Additionally, the contents of non-enzymatic antioxidant compounds, namely total phenolics (TP), flavonoids, ascorbic acid (AsA), and reduced glutathione (GSH), were quantitatively analyzed. All the aforementioned parameters were determined using commercial assay kits provided by Suzhou Comin Biotechnology Co., Ltd. (Suzhou, China), strictly in accordance with the manufacturer’s instructions. Detailed kit information is provided in Table 1.

### 2.3. Lipidomic Analysis

#### 2.3.1. Chemicals and Reagents

Among the liquid chromatography–tandem mass spectrometry (LC-MS) grade reagents used in this study, methanol was purchased from Merck (Darmstadt, Germany). Acetonitrile and isopropanol were obtained from Shanghai Xingke High-Purity Solvents Co., Ltd. (Shanghai, China). Formic acid was sourced from Sigma-Aldrich (St. Louis, MO, USA). Ammonium formate and chloroform were provided by Fisher Scientific (Waltham, MA, USA), and methyl tert-butyl ether (MTBE) was acquired from CNW Technologies (Düsseldorf, Germany). Ultrapure water was prepared using a laboratory ultrapure water system (Merck, Darmstadt, Germany).

#### 2.3.2. Sample Preparation and Lipid Extraction

Initially, all harvested mycelial samples were lyophilized and ground into a fine powder. A quantitative amount of the dried sample was weighed into a centrifuge tube, and a lipid extraction solvent (MTBE:methanol = 3:1, *v*/*v*) was added. The mixture was vortexed at 2500 rpm at room temperature for disruption and extraction. Subsequently, ultrapure water was added to the system to induce the polar phase separation of the aqueous and organic layers. Following centrifugation, the upper lipid-rich organic phase was carefully collected and evaporated to dryness. Finally, the dried extract was reconstituted with a lipid reconstitution solvent (acetonitrile:isopropanol = 1:1, *v*/*v*). After a further centrifugation step, the clear supernatant was transferred into an autosampler vial for LC-MS/MS data acquisition.

#### 2.3.3. Chromatographic and Mass Spectrometry Conditions

Lipidomic data acquisition was performed using an ultra-performance liquid chromatography (UPLC) system coupled with a tandem mass spectrometer (MS/MS).

Liquid chromatography conditions were as follows:

(1) Column: Thermo Accucore™ C30 column (2.6 μm, 2.1 mm × 100 mm i.d., Thermo Fisher Scientific, Waltham, MA, USA);
(2) Mobile phases: phase A, acetonitrile/water (60:40, *v*/*v*) containing 0.1% formic acid and 10 mmol/L ammonium formate; phase B, acetonitrile/isopropanol (10:90, *v*/*v*) containing 0.1% formic acid and 10 mmol/L ammonium formate;
(3) Gradient elution program: 0 min, A/B = 80:20 (*v*/*v*); 2 min, 70:30 (*v*/*v*); 4 min, 40:60 (*v*/*v*); 9 min, 15:85 (*v*/*v*); 14 min, 10:90 (*v*/*v*); 15.5 min, 5:95 (*v*/*v*); 17.3 min, 5:95 (*v*/*v*); 17.5 min, 80:20 (*v*/*v*); and 20 min, 80:20 (*v*/*v*);
(4) Flow rate: 0.35 mL/min; column temperature: 45 °C; injection volume: 2 μL.

Mass spectrometry conditions were as follows:

The temperature of the electrospray ionization (ESI) source was set at 500 °C. The ion spray voltage was 5500 V in positive ion mode and −4500 V in negative ion mode. Ion source gas 1 (GS1) was set at 45 psi, gas 2 (GS2) at 55 psi, and curtain gas (CUR) at 35 psi. In the triple quadrupole system, each ion pair was scanned and detected based on the optimized declustering potential (DP) and collision energy (CE).

#### 2.3.4. Lipid Identification and Quantification

Lipid identification was based on comparison with mass spectral library, and qualitative analysis was performed according to the retention time (RT) and precursor/product ion pair information of the detected compounds. Lipid quantification was carried out using multiple reaction monitoring (MRM) on a triple quadrupole mass spectrometer (SCIEX, Framingham, MA, USA). After obtaining the lipid mass spectrometric data from different samples, the chromatographic peak areas of all detected compounds were integrated, and quantitative analysis was performed using the internal standard method, rather than being calculated on total lipid amount in% or dry mass. The internal standards used were a dedicated mixed isotopic standard for lipidomics. The detailed information on the internal standards is provided in Table S1 of the Supplementary Materials.

### 2.4. Data Preprocessing and Statistical Analysis

Antioxidant data were analyzed by one-way ANOVA after normality testing using SAS software (Version 9.4). Raw lipidomics mass spectrometry data were acquired and processed with Analyst 1.6.3, including baseline correction, peak detection, integration, retention time correction, alignment, and normalization. Regarding multivariate statistical analysis, Principal Component Analysis (PCA) was initially employed to evaluate the distribution and separation trends of the lipidomes among the different groups. Subsequently, an Orthogonal Partial Least Squares Discriminant Analysis (OPLS-DA) model was utilized to maximize the segregation between different pH groups. The screening criteria for differentially accumulated lipids (DALs) were set at a Variable Importance in Projection (VIP) score > 1.0 and a *p*-value < 0.05. Finally, Hierarchical Cluster Analysis (HCA) and KEGG pathway enrichment analysis were conducted to explore the remodeling patterns of lipid metabolism in the mycelia under various pH conditions.

## 3. Results

### 3.1. Antioxidant Activity

**Figure 1:**
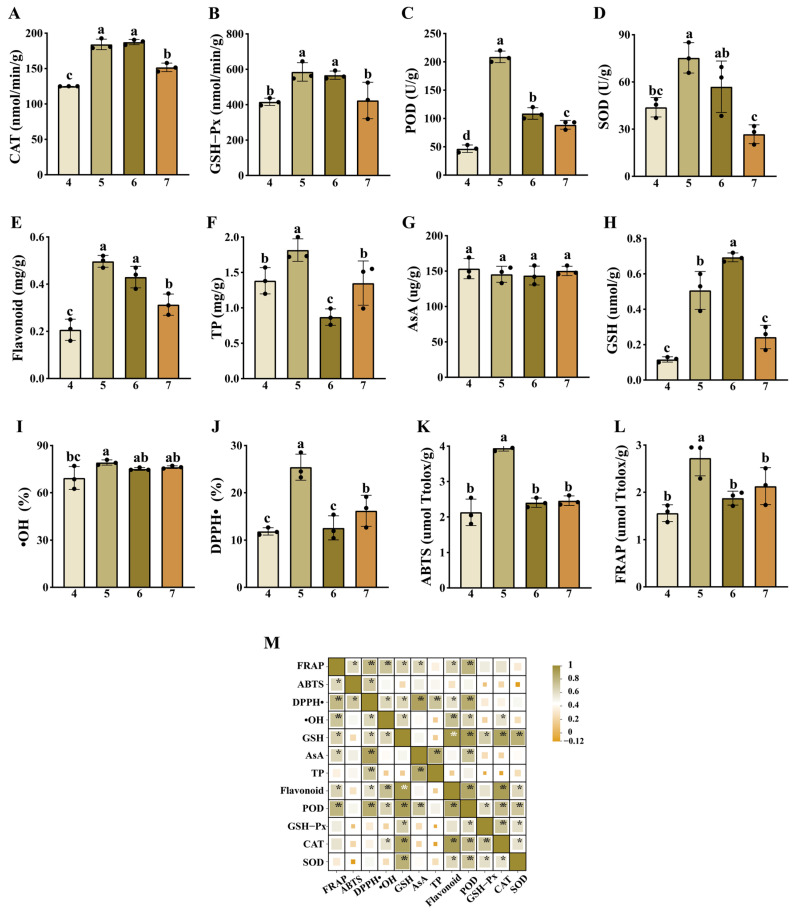
Comparison of the antioxidant capacities of *S. hepiali* cultured under different initial pH conditions. (**A**) Catalase (CAT) activity; (**B**) Glutathione peroxidase (GSH-Px) activity; (**C**) Peroxidase (POD) activity; (**D**) Superoxide dismutase (SOD) activity; (**E**) Flavonoid content; (**F**) Total phenolic (TP) content; (**G**) Ascorbic acid (AsA) content; (**H**) Reduced glutathione (GSH) content; (**I**) Hydroxyl radical (•OH) scavenging rate; (**J**) 2,2-diphenyl-1-picrylhydrazyl radical (DPPH•) scavenging rate; (**K**) Total antioxidant capacity (ABTS assay); (**L**) Total antioxidant capacity (FRAP assay); (**M**) Correlation analysis of the evaluated indicators. * indicates *p *< 0.05, ** indicates *p *< 0.01. Different lowercase letters indicate significant differences between the treatment groups (one-way ANOVA, *p* < 0.05). The *x*-axis represents the initial pH of the culture medium.

The antioxidant responses of *S. hepiali* under different initial pH conditions were systematically evaluated using 12 physicochemical indicators. Among the four antioxidant enzyme indicators, the activities of POD, SOD, and GSH-Px all peaked at pH 5, recording values of 208.29 ± 5.88 U/g, 75.31 ± 5.59 U/g, and 585.43 ± 30.09 nmol/min/g, respectively. These values were significantly higher than those in the pH 4 and pH 7 groups (*p* < 0.05). Overall, the activities of CAT, POD, SOD, and GSH-Px exhibited an initial increase followed by a subsequent decrease as the initial pH of the medium increased. Regarding the non-enzymatic antioxidant system, the contents of flavonoids and TP, as well as the scavenging rates of DPPH• and •OH, also peaked at pH 5. Conversely, the GSH content reached its maximum at pH 6 (0.69 ± 0.014 μmol/g). No significant differences in AsA content were observed among groups (*p* > 0.05). The total antioxidant capacity at pH 5 was significantly stronger than that of the other three treatment groups (*p* < 0.05), among which no significant differences were detected. Correlation analysis further indicated that CAT and SOD activities were positively associated with GSH and flavonoid levels, as well as the activities of POD and GSH-Px. Furthermore, the total antioxidant capacity (evaluated via FRAP and ABTS assays) exhibited strong positive synergistic relationships with the DPPH• and •OH scavenging rates, alongside the levels of GSH, AsA, flavonoids, and POD (Figure 1).

### 3.2. Compositional Profiling of the S. hepiali Lipidome Under Different pH Conditions

**Figure 2:**
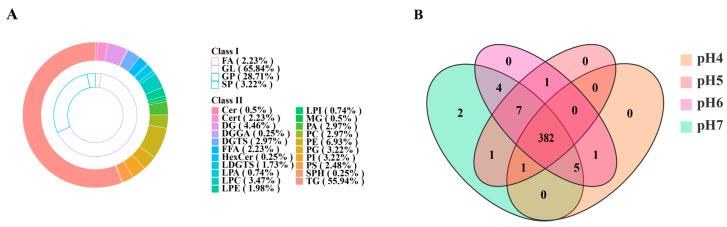
Lipid identification in *S. hepiali*. (**A**) Lipid classification; (**B**) Venn diagram of identified lipids across the four sample groups.

A total of 404 lipid molecular species, spanning 21 subclasses, were identified across the four groups (Figure 2A; Table S2). In terms of relative abundance, glycerolipids and glycerophospholipids dominated the lipidomic profile. The most abundant lipid classes were triglycerides (TG, 55.94%), phosphatidylethanolamines (PE, 6.93%), diglycerides (DG, 4.46%), phosphatidylglycerols (PG, 3.22%), phosphatidylinositols (PI, 3.22%), and phosphatidylcholines (PC). Venn diagram analysis (Figure 2B) revealed that 382 lipid species were shared among all groups. This suggests that environmental pH did not fundamentally alter the basic architecture of the fungal lipidome but mainly influenced the accumulation levels of specific lipid molecules.

### 3.3. Multivariate Statistical Analysis of the Lipidomic Profiles

**Figure 3:**
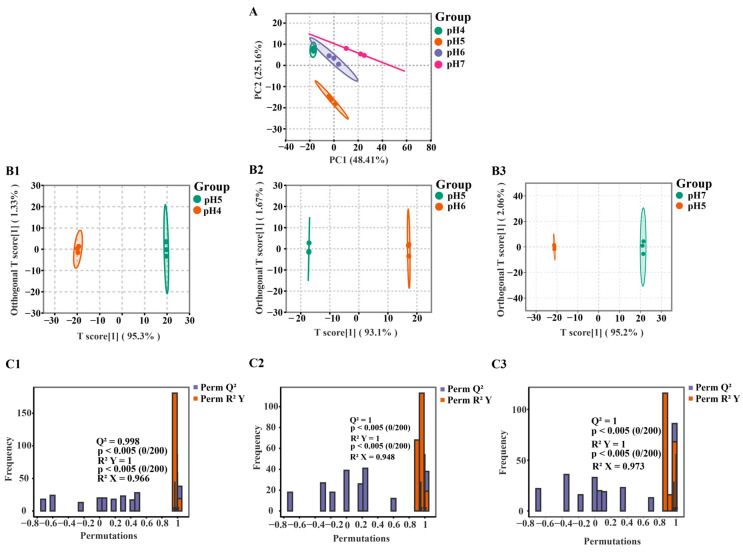
(**A**) PCA score plot. PC1 and PC2 represent the variances explained by the first and second principal components, respectively; (**B1**–**B3**) OPLS-DA score plots for the comparisons of pH 4 vs. pH 5, pH 6 vs. pH 5, and pH 7 vs. pH 5, respectively; (**C1**–**C3**) OPLS-DA validation (permutation test) plots for pH 4 vs. pH 5, pH 6 vs. pH 5, and pH 7 vs. pH 5, respectively. The vertical axis displays the frequency of the model classification performance over 200 random permutations, and the horizontal axis represents the R2 and Q2 values of the models.

Unsupervised PCA was initially employed to visualize the overall variance among the groups. In the PCA score plot (Figure 3A), PC1 and PC2 explained 48.41% and 25.16% of the total variance, respectively, accounting for 73.57% cumulatively. Furthermore, the four sample groups showed clear and distinct clustering patterns. Biological replicates within each group clustered tightly, confirming the reliability of the experimental procedures and the stability of the instrumental analysis.

OPLS-DA was then used to further characterize intergroup differences. Pairwise comparisons of pH 4 vs. pH 5, pH 6 vs. pH 5, and pH 7 vs. pH 5 (Figure 3(B1–B3)) all showed clear separation in the score plots, suggesting substantial lipid metabolic variation under different pH conditions. To validate the robustness and predictive reliability of the models, permutation tests with 200 iterations were performed. The validation results (Figure 3(C1–C3)) were as follows: for the pH 4 vs. pH 5 group, R^2^X = 0.966, R^2^Y = 1, Q^2^ = 0.998; for the pH 6 vs. pH 5 group, R^2^X = 0.948, R^2^Y = 1, Q^2^ = 1; and for the pH 7 vs. pH 5 group, R^2^X = 0.973, R^2^Y = 1, Q^2^ = 1. Together with permutation test *p* values < 0.005, these results indicate that the models were robust and showed no obvious overfitting.

### 3.4. Screening and Trend Analysis of Differentially Accumulated Lipids (DALs)

**Figure 4:**
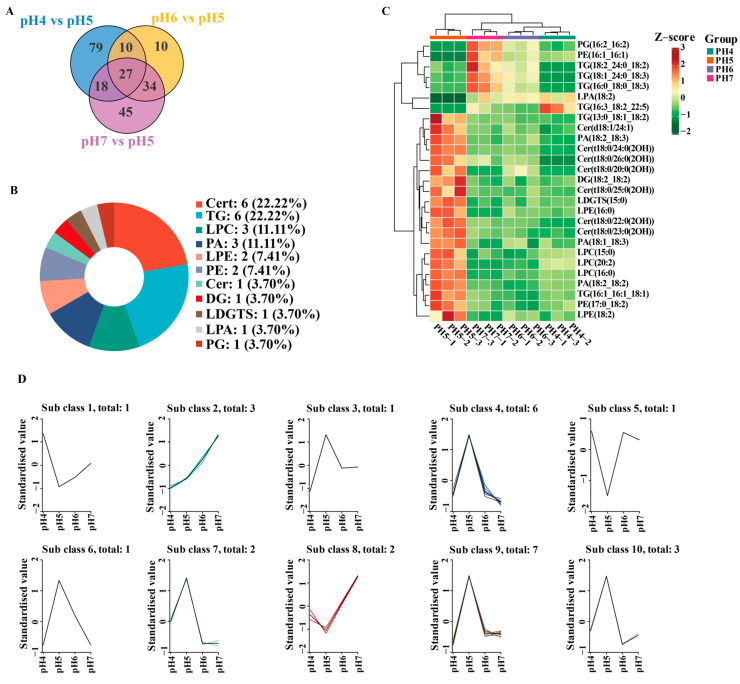
Analysis of the differentially accumulated lipids (DALs) in *S. hepiali* under different pH conditions. (**A**) Venn diagram of the DALs among the pH 4 vs. pH 5, pH 6 vs. pH 5, and pH 7 vs. pH 5 comparison groups; (**B**) Pie chart illustrating the subclass distribution of the shared DALs; (**C**) Hierarchical clustering heatmap of the shared DALs; (**D**) K-means clustering analysis of the shared DALs. The *x*-axis represents the sample groups, and the *y*-axis indicates the standardized relative lipid abundances.

Based on the Variable Importance in Projection (VIP) scores derived from the OPLS-DA model, combined with significant differences from univariate analysis, differentially accumulated lipids (DALs) were screened using VIP > 1.0 and *p* < 0.05. A Venn diagram analysis of the pH 4 vs. pH 5, pH 6 vs. pH 5, and pH 7 vs. pH 5 comparisons identified 27 core DALs that were commonly regulated across all groups. These shared DALs were broadly distributed across 11 distinct lipid subclasses. Among them, phytoceramides (Cert, 22.22%) and TG (22.22%) accounted for the highest proportions, followed by lysophosphatidylcholines (LPC, 11.11%) and phosphatidic acids (PA, 11.11%) (Figure 4A,B). Additionally, other subclasses included lysophosphatidylethanolamines (LPE), PE, ceramides (Cer), and DG. Hierarchical clustering showed clear differences in DAL abundance among the four groups. Notably, samples from the pH 5 group clustered independently into a single clade. Within this group, the majority of the differential lipids exhibited significantly high expression levels (indicated by red areas). Conversely, the contents of these lipids generally decreased as the pH value decreased (pH 4) or increased (pH 6 and pH 7).

K-means clustering analysis was employed to categorize the dynamic variation trends of the 27 shared DALs across the four pH gradients (Figure 4D). Among the sub-classes, a total of 17 DALs from sub-class 3 (*n* = 1), sub-class 4 (*n* = 6), sub-class 6 (*n* = 1), sub-class 7 (*n* = 2), sub-class 9 (*n* = 7), and sub-class 10 (*n* = 3) exhibited a consistent trend of their contents peaking at pH 5 and subsequently declining rapidly. In contrast, the contents of the DALs in sub-class 2 (*n* = 3) displayed a trend of gradual accumulation with increasing pH.

**Figure 5:**
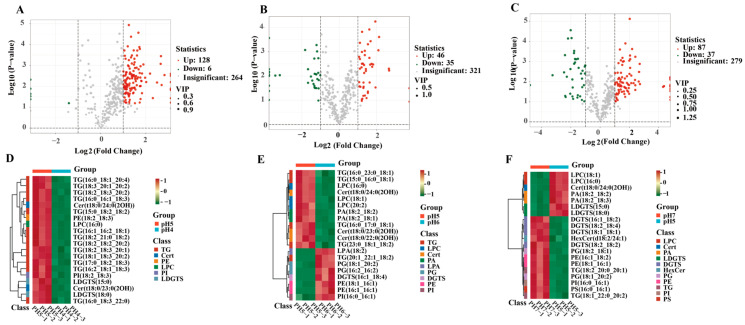
Analysis of environmental pH-driven differential lipid metabolism in *S. hepiali*. (**A**–**C**) Volcano plots for the pH 4 vs. pH 5, pH 6 vs. pH 5, and pH 7 vs. pH 5 comparison groups, respectively. Red dots indicate significantly up-regulated lipids, whereas green dots represent significantly down-regulated lipids; (**D**–**F**) Hierarchical clustering heatmaps of the top 20 differential lipids in each comparison group. The color scale from green to red indicates relative lipid abundances ranging from low to high.

Volcano plots results indicated that changes in environmental pH induced a significant reorganization of the fungal lipidome. In the pH 4 vs. pH 5 comparison, 134 differential lipids were identified, comprising 128 significantly up-regulated and 6 significantly down-regulated lipids (Figure 5A). By comparison, in the pH 6 vs. pH 5 group, 81 differential lipids were screened, including 46 up-regulated and 35 down-regulated lipids (Figure 5B). Furthermore, in the pH 7 vs. pH 5 group, 124 differential lipids were identified, consisting of 87 up-regulated and 37 down-regulated lipids (Figure 5C). HCA was performed on the top 20 differential lipids in each comparative group. The clustering heatmaps (Figure 5D–F) clearly revealed the specific distribution patterns of lipid abundances in response to environmental pH changes. Across the three comparative groups, the core differential lipids consistently exhibited a highly specific enrichment pattern at pH 5. These lipids accumulated at relatively high levels (red scale) under pH 5 conditions, whereas their relative contents were significantly reduced (green scale) at pH 4, 6, and 7.

### 3.5. KEGG Pathway Enrichment Analysis of Differentially Accumulated Lipids

**Figure 6:**
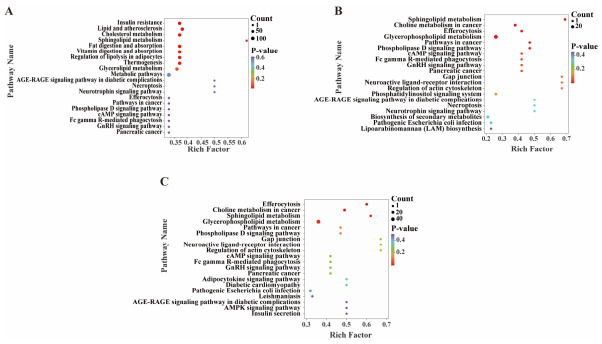
KEGG pathway enrichment analysis of the differential lipids. (**A**) pH 4 vs. pH 5; (**B**) pH 6 vs. pH 5; (**C**) pH 7 vs. pH 5. The *x*-axis represents the enrichment factor corresponding to each pathway, and the *y*-axis represents the pathway names (sorted by *p*-value). The color of the dots reflects the *p*-value, with redder colors indicating a higher significance of enrichment.

**Figure 7:**
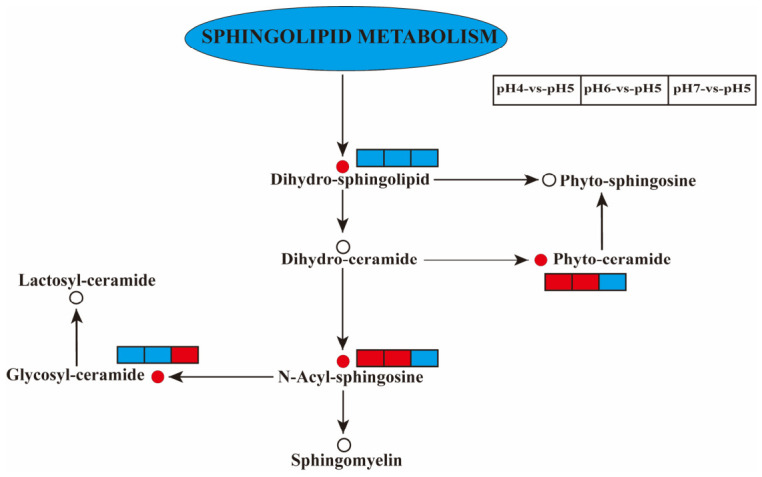
Overview of the metabolic network illustrating the responses of key lipid molecules in the sphingolipid metabolism pathway to pH variation. Red circles indicate significantly altered metabolites; white circles represent undetected metabolites; red rectangles denote significantly up-regulated metabolites between groups; blue rectangles denote significantly down-regulated metabolites between groups; and solid arrows represent promotive effects.

To further elucidate the biological functions and potential underlying mechanisms by which environmental pH regulates lipid metabolism, the identified DALs were mapped to the Kyoto Encyclopedia of Genes and Genomes (KEGG) database for pathway enrichment analysis. The top 20 enriched pathways were selected for each comparison group. In the pH 4 vs. pH 5 comparison (Figure 6A), pathways including sphingolipid metabolism, cholesterol metabolism, and lipid and atherosclerosis exhibited significant enrichment. Similarly, in both the pH 6 vs. pH 5 and pH 7 vs. pH 5 groups (Figure 6B,C), the sphingolipid metabolism, choline metabolism in cancer, efferocytosis, and glycerophospholipid metabolism pathways were significantly enriched. Among these pathways, sphingolipid metabolism was the only pathway commonly enriched across all three comparisons. As illustrated in Figure 7, pH variation induced marked changes in several key intermediates within the sphingolipid metabolic network. Specifically, the abundances of dihydrosphingolipids, N-acylsphingosines, phytoceramides, and glycosylceramides were notably altered, indicating that sphingolipid metabolism may play a central role in the adaptation of *S. hepiali* to environmental acid–base variation.

## 4. Discussion

Previous studies have demonstrated that changes in culture pH can regulate the levels of antioxidant enzymes, including SOD, CAT, and POD, as well as non-enzymatic antioxidant components, such as phenolic compounds, thereby influencing free radical scavenging activity, reducing power, and metal-chelating capacity [17,26]. Our results indicate that environmental pH significantly regulates the antioxidant capacity of *S. hepiali*. The activities of core antioxidant enzymes such as POD, SOD, and GSH-Px and the contents of key non-enzymatic metabolites like flavonoids and TP all peaked at pH 5. This conclusion is further supported by total antioxidant capacity evaluations (FRAP and ABTS). These findings suggest that a slightly acidic environment pH 5 may be the optimal condition to activate the antioxidant potential of *S. hepiali*. Similar observations have been reported in other fungi, in which the initial fermentation pH profoundly influenced secondary metabolism and antioxidant activity. Specifically, altering the medium pH was proven to induce an increase in the antioxidant activity of *Ganoderma* [18]. *Cordyceps militaris* mycelia exhibited higher antioxidant capacity at an initial growth pH of 8–9 [27]. The extracellular polysaccharides of *Tremella fuciformis* cultured under pH 9 conditions showed greater reducing power and stronger DPPH radical scavenging activity [28].

While the core of metabolomics research lies in polar metabolites such as amino acids, organic acids, and nucleotides [29], lipidomics focuses on the comprehensive qualitative and quantitative analysis of diverse lipid molecules, having evolved into an independent scientific discipline [30,31]. According to the classification standards of the LIPID MAPS system, lipids are categorized into eight major classes: fatty acyls, glycerolipids, glycerophospholipids, sterol lipids, prenol lipids, sphingolipids, saccharolipids, and polyketides [32,33,34]. Using lipidomics, a total of 404 lipid compounds were identified in *S. hepiali*. These lipids were mainly assigned to four major categories: glycerolipids, glycerophospholipids, sphingolipids, and fatty acyls. Among them, TG, PE, and DG constituted the core components of the *S. hepiali* lipidome, and TG is the principal neutral lipid for energy storage in eukaryotic cells [35,36,37].This profile is fundamentally consistent with the lipidomic characteristics of *O. Sinensis* [38].

Multivariate statistical analysis confirmed that different pH gradients drove a drastic systemic remodeling of the fungal lipidomic profile. KEGG enrichment analysis revealed that, whether the environmental pH decreased from 5 to 4 or increased from 5 to 6/7, sphingolipid metabolism was the sole shared core pathway significantly enriched across all three comparative groups. Sphingolipids are not merely structural supporting components of the plasma membrane; they are core bioactive molecules governing apoptosis, and cellular growth [39,40]. Bondarenko, S.A. reported that changes in environmental pH rapidly increased the proportions of sphingolipids and sterols in the plasma membrane. This finding suggests that pH variation can induce remodeling of sphingolipid-related membrane structures [23]. Sasaki, H. confirmed that pH can strongly affect the aggregation behavior of sphingosine by altering ionic interactions and hydrogen-bonding states [41]. This, in turn, may influence sphingolipid metabolism. Our results demonstrated profound fluctuations within the sphingolipid metabolic network in response to pH variation, wherein dihydrosphingolipids, N-acylsphingosines, phytoceramides, and glycosylceramides all underwent significant directional alterations. Ceramides are crucial regulatory molecules in signal transduction [42]. pH changes can regulate the activity of ceramidase. As a result, the hydrolysis of intracellular ceramide is altered, which is important for cellular responses [43].

On the one hand, ROS can regulate sphingolipid metabolism by modulating enzymes involved in ceramide and sphingosine-1-phosphate (S1P) synthesis. The balance between ROS production and antioxidant defense determines the metabolic direction of the ceramide–S1P axis. In turn, the homeostasis of ceramide and S1P can regulate multiple ROS-mediated signaling pathways [44]. On the other hand, a study by Kajiwara et al. demonstrated that intracellular sphingolipids can regulate ROS levels [45]. Furthermore, Niles et al. found that while intracellular sphingolipids effectively inhibit ROS, the ROS generated from their depletion can act as key signaling molecules to create a positive feedback loop that regulates sphingolipid biosynthesis [46]. In the present study, *S. hepiali* exhibited the highest antioxidant activity at pH 5, and sphingolipid metabolism was the universally enriched pathway under all conditions. These outcomes may indicate that the pH 5 environment may induce intracellular ROS accumulation in *S. hepiali*. This ROS accumulation initiates the antioxidant potential of the fungus, activating the expression of antioxidant enzymes while promoting the synthesis of antioxidants like flavonoids. Meanwhile, ROS acts as a signaling factor to regulate sphingolipid metabolism. This occurs by regulating the synthesis of key lipids such as ceramides and sphingosines, thereby modulating ROS levels. Collectively, this study provides an essential theoretical basis and practical insights for optimizing the industrial fermentation process of *S. hepiali* fungi.

## 5. Conclusions

This study elucidated the metabolic regulatory effects of the initial culture pH on *S. hepiali* from the dual perspectives of antioxidant phenotypes and the lipidome. It was established that a slightly acidic environment (pH 5) serves as the optimal condition for activating the antioxidant defense system, promoting the accumulation of both enzymatic and non-enzymatic antioxidants in the mycelia. Lipidomic profiling revealed that the dominant lipid subclasses in *S. hepiali* include TG, PE, and DG, with TG accounting for over 50% of the total lipid content. Using the pH 5 group as a reference, 134, 81, and 124 DALs were identified in the pH 4, pH 6, and pH 7 groups, respectively. Moreover, KEGG pathway analysis highlighted that sphingolipid metabolism was the sole universally enriched pathway across all comparisons. Overall, these results provide a theoretical foundation for optimizing submerged fermentation to enhance antioxidant-related bioactive compounds and lipid quality in *Cordyceps*.

## Figures and Tables

**Table 1:** The exact names and catalog numbers of the aforementioned kits.

Measured Indicators	Full Name	Catalog Number
CAT	Catalase (CAT) assay kit	CAT-2-Y
GPX-Px	glutathione peroxidase (enzymatic method) assay kit	GPX-2-W
POD	peroxidase (POD) assay kit	GPX-2-W
SOD	superoxide dismutase (SOD) assay kit	SOD-2-Y
TP	total phenolics (TP) assay kit	TP-2-G
flavonoids	Plant flavonoid assay kit	LHT-2-G
AsA	reduced ascorbic acid (AsA) content assay kit	ASA-2-W
GSH	reduced glutathione (GSH) assay kit	GSH-2-W
total antioxidant capacity (ABTS)	total antioxidant capacity (ABTS) assay kit	ABTS-2-D
total antioxidant capacity (FRAP)	total antioxidant capacity (FRAP) assay kit	FRAP-2-G
the scavenging rates of •OH	hydroxyl radical scavenging capacity assay kit	QZQ-2-G
the scavenging rates of DPPH•	total antioxidant capacity (DPPH method) assay kit	DPPH-1-D

## Data Availability

The original contributions presented in this study are included in the article/Supplementary Material. Further inquiries can be directed to the corresponding authors.

## Supplementary Materials

The following supporting information can be downloaded at: https://www.mdpi.com/article/10.3390/jof12050367/s1, Table S1: Internal standard information for quantitative lipids; Table S2: Identified lipid molecular information.

## Abbreviations

The following abbreviations are used in this manuscript:

ABTS	2,2′-azinobis(3-ethylbenzothiazoline-6-sulfonic acid)
AsA	Ascorbic acid
CAT	Catalase
CER	Ceramides
CERT	Phytoceramides
DALs	Differentially accumulated lipids
DG	Diglycerides
DPPH•	2,2-diphenyl-1-picrylhydrazyl radical
FRAP	Ferric reducing antioxidant power
GSH	Glutathione
GSH-Px	Glutathione peroxidase
HCA	Hierarchical Cluster Analysis
KEGG	Kyoto Encyclopedia of Genes and Genomes
LPC	Lysophosphatidylcholines
LPE	Lysophosphatidylethanolamines
MTBE	Methyl tert-butyl ether
*O. sinensis*	*Ophiocordyceps sinensis*
OPLS-DA	Orthogonal Partial Least Squares Discriminant Analysis
PA	Phosphatidic acids
PCA	Principal Component Analysis
PC	Phosphatidylcholines
PE	Phosphatidylethanolamines
PG	Phosphatidylglycerols
PI	Phosphatidylinositols
POD	Peroxidase
ROS	Reactive oxygen species
*S. hepiali*	*Samsoniella hepiali*
S1P	sphingosine-1-phosphate
SOD	Superoxide dismutase
TG	Triglycerides
TP	Total phenolics
UPLC-MS/MS	Ultra-performance liquid chromatography-tandem mass spectrometry
VIP	Variable Importance in Projection
•OH	Hydroxyl radicals
